# Reverse-switching radiative cooling for synchronizing indoor air conditioning

**DOI:** 10.1515/nanoph-2023-0699

**Published:** 2024-02-01

**Authors:** Yang Liu, Yi Zheng

**Affiliations:** Department of Mechanical and Industrial Engineering, Northeastern University, Boston, MA 02115, USA; Department of Chemical Engineering, Northeastern University, Boston, MA 02115, USA

**Keywords:** reverse radiative cooling, phase-change material, indoor air conditioning, synchronized heating and cooling, precise temperature control

## Abstract

Switchable radiative cooling based on the phase-change material vanadium dioxide (VO_2_) automatically modulates thermal emission in response to varying ambient temperature. However, it is still challenging to achieve constant indoor temperature control solely using a VO_2_-based radiative cooling system, especially at low ambient temperatures. Here, we propose a reverse-switching VO_2_-based radiative cooling system, assisting indoor air conditioning to obtain precise indoor temperature control. Unlike previous VO_2_-based radiative cooling systems, the reverse VO_2_-based radiative cooler turns on radiative cooling at low ambient temperatures and turns off radiative cooling at high ambient temperatures, thereby synchronizing its cooling modes with the heating and cooling cycles of the indoor air conditioning during the actual process of precise temperature control. Calculations demonstrate that our proposed VO_2_-based radiative cooling system significantly reduces the energy consumption by nearly 30 % for heating and cooling by indoor air conditioning while maintaining a constant indoor temperature, even surpassing the performance of an ideal radiative cooler. This work advances the intelligent thermal regulation of radiative cooling in conjunction with the traditional air conditioning technology.

## 1 Introduction

In recent years, energy consumption has shown a steady rise alongside rapid economic development [1]. Heating and cooling systems in buildings are pivotal in providing a comfortable indoor environment, contributing to approximately 40 % of the total energy consumption in these structures [2], [3]. Consequently, it is crucial to prioritize the development of energy-efficient thermal technologies for energy conservation applications [4]. Take cooling for example, passive radiative cooling without energy consumption has emerged as an appealing approach for improving building heat dissipation by radiating heat from cooling materials installed on exterior walls into the ultracold outer space through the atmospheric transparent window (8–13 μm) and reflecting sunlight (0.3–2.5 μm) at the same time[5], [6], [7], [8], [9], [10], [11], [12], [13], [14], [15]. To obtain high-performance radiative cooling, a variety of cooing materials and structures with high solar reflectance (*R*_solar_, *λ* ∼ 0.3–2.5 μm) and high infrared emissivity (*ε*_ir_, *λ* ∼ 8–13 μm) within the atmospheric transparent window have been proposed, including bio-inspired structures [16], [17], [18], [19], [20], film-based structures [21], [22], pigmented paint films [23], [24], [25], particle-based structures [24], [25], [26], [27], [28], as well as multilayer-film [5], [29], [30], [31], [32] and patterned-surface photonic structures [33], [34], [35], [36], [37].

Although these radiative cooling structures exhibit highly efficient all-day cooling performance due to the static spectral characteristics, their fixed radiative cooling can cause overcooling effects on cold days, thereby resulting in additional heating costs. In response to the changing ambient temperature, some research has focused on switchable radiative cooling systems utilizing the phase change material vanadium dioxide (VO_2_) to avoid the overcooling consequences [38], [39], [40], [41], [42]. In these studies, VO_2_-based radiative coolers can automatically modulate their infrared emissivity based on the phase change of VO_2_, where the radiative coolers enhance the infrared emissivity when their temperature is higher than the critical temperature and suppress the infrared emissivity when their temperature is below the critical temperature. The critical temperature depends on the phase-change temperature of VO_2_, which can be adjusted by doping other elements into pure VO_2_ [43]. First, Ono et al. proposed a concept of self-adaptive radiative cooling based on the combination of VO_2_-based multilayer cooler and the top spectrally selective filter [38]. After that, Kim [39], Zhang [40], Kort-Kamp [41], and Liu [42] have successively proposed similar switchable radiative cooling systems based on the VO_2_ photonic structures, all of which achieve radiative cooling at high ambient temperatures and keep their temperature around the ambient temperature at low temperatures. In addition, other types of switching radiative cooling systems also rely on mechanical switches [44], [45], [46], [47], [48], elastomeric modulators [49], [50], and temperature-sensitive material switches [51], [52], [53]. In fact, radiative cooling technology can only theoretically control the temperature of the cooling materials and cannot directly affect the indoor temperature due to a range of influencing factors, including wall thermal transfer, indoor ventilation, indoor lighting, indoor air conditioning, etc. In addition, achieving precise control of a constant indoor temperature becomes more challenging when relying solely on radiative cooling technology. Despite its potential to reduce energy consumption of indoor temperature regulation, passive radiative cooling technology has not yet reached a point where it can fully replace active air conditioning. Therefore, it is crucial to investigate the auxiliary effects of passive radiative cooling when combined with active indoor air conditioner–based temperature regulation, especially in terms of energy saving.

In this paper, we propose a VO_2_-based radiative cooling system with the automatic reverse switching function that works in conjunction with the heating and cooling modes of indoor air conditioning to maintain a constant indoor temperature. Compared to conventional VO_2_-based switching cooling structures, the reverse VO_2_-based thermal system reduces its infrared emissivity (0.20) when its temperature exceeds the set temperature and increases the infrared emissivity (0.80) when its temperature falls below the set temperature, which is more suitable for use in conjunction with machine-based indoor air conditioning. Our proposed VO_2_-based radiative cooling system synchronizes its cooling modes with the heating and cooling cycles of the indoor air conditioning during the actual process of precise temperature control. Additionally, we quantify the energy-saving potential of VO_2_-based radiative technology in the precise control of indoor temperature. As a result, this work demonstrates the combination of the passive VO_2_-based radiative thermal technology and the active air conditioning technology can pave the way for applications of precise indoor temperature control with significant energy-saving advantages.

## 2 Theoretical model and methods

**Figure 1:**
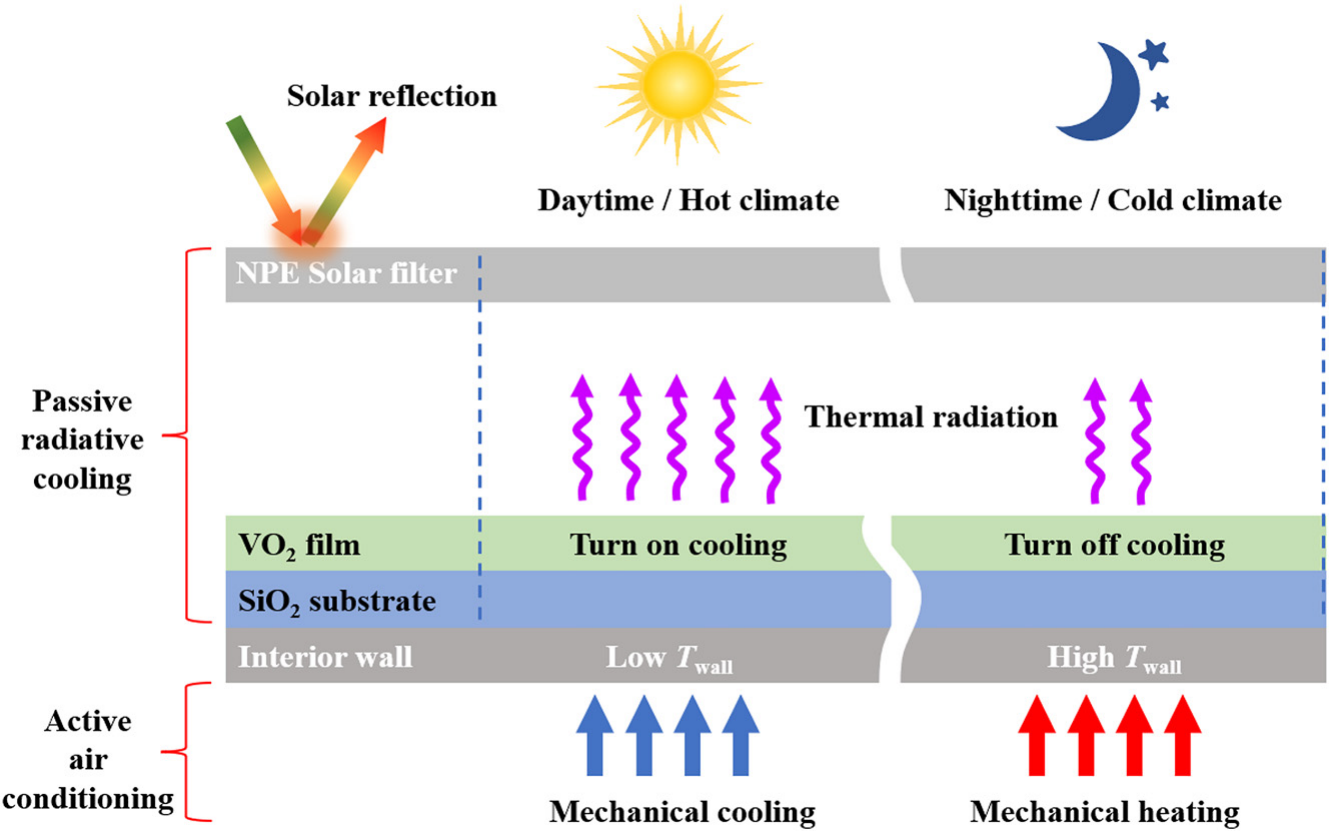
Design of the precise temperature control system that combines active indoor air conditioning and passive radiative cooling, where passive radiative cooling component consists of the top NPE and the bottom VO_2_-based radiator.

Here, we propose a temperature control system integrating both active indoor air conditioning and passive radiative cooling, as schematically depicted in Figure 1. The passive radiative cooling component consists of the nanoporous polyethylene (NPE) [54] working as the top solar filter and the bottom VO_2_-based double-layer film radiator. The structural parameters (thickness *h*_NPE_ = 12 μm and pore size *R*_pore_ = 400 nm) and infrared and visible transmittances of NPE can be found in the literature [54]. The bottom radiator consists of a VO_2_ thin film (*h*_1_ = 150 nm) deposed on a silicon dioxide (SiO_2_) substrate with a thickness of 500 µm, tightly attached to the wall. More significantly in this work, the VO_2_-based radiative cooling component in the entire system serves as an auxiliary element for precise temperature control, aiming at reducing the energy consumption of air conditioning. The active indoor air conditioning component in Figure 1 adjusts its cooling and heating power according to the set of indoor temperature, playing a pivotal role in achieving precise temperature control.

**Figure 2:**
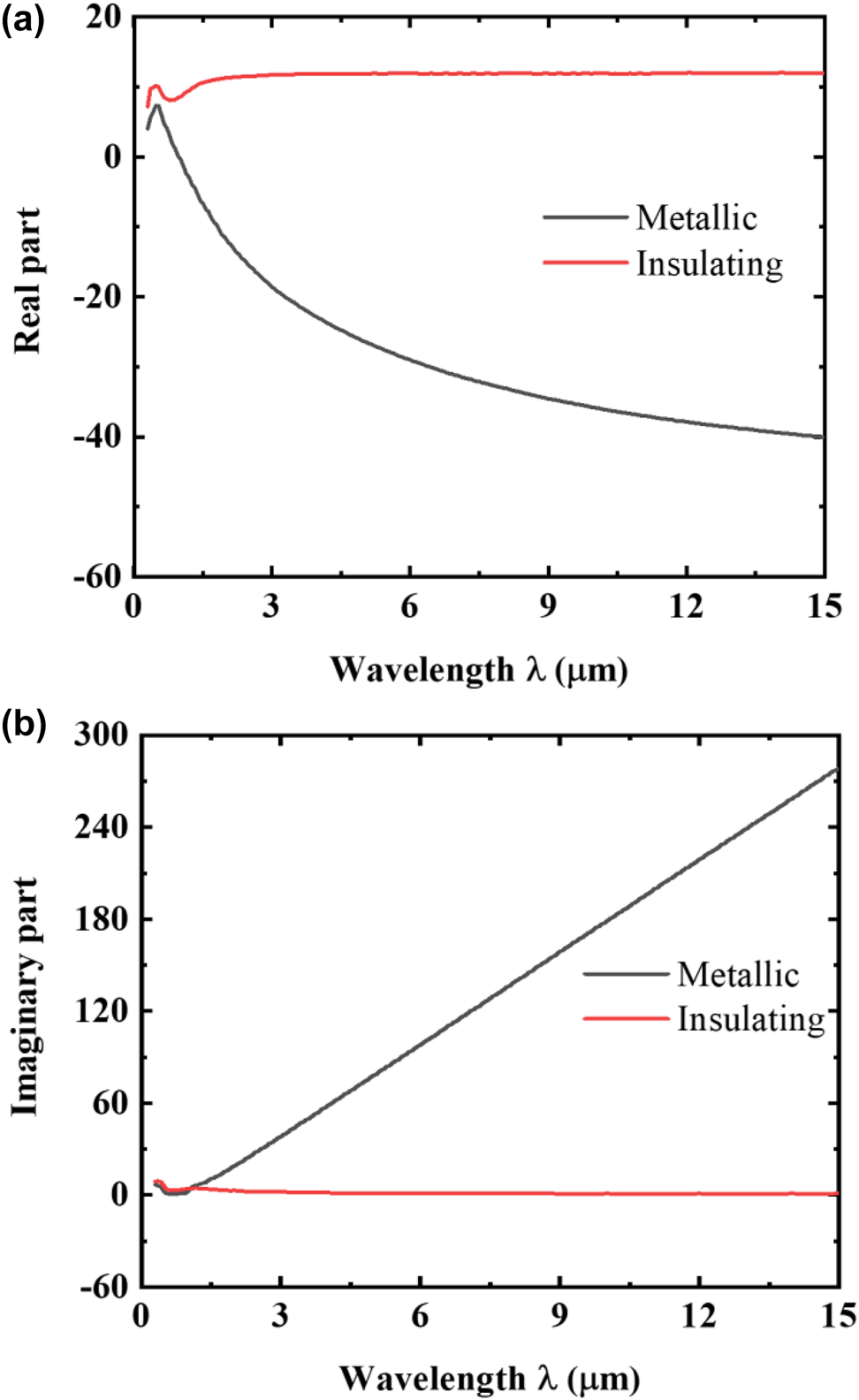
Optical properties of VO_2_ at metallic and insulating states. (a) Real part and (b) imaginary part of VO_2_ permittivity within the wavelength range (0.3–15 μm).

Pure VO_2_, a phase-change material, can achieve a reversible insulator-to-metal transition around 68 °C [55]. When the temperature of VO_2_ is above the critical temperature *T*_c_, VO_2_ exhibits metallic behavior and changes to insulating state when its temperature falls below *T*_c_. However, to broaden the application of VO_2_’s phase-change property, its *T*_c_ has been adjusted by doping other elements, such as molybdenum (Mo), tungsten (W), etc., into pure VO_2_ to approach to the room temperature [43]. In this study, the permittivity of both metallic and insulating VO_2_ during the wavelength range of 0.3 μm–15 μm is presented in Figure 2 [38]. Meanwhile, the phase-change process of VO_2_ with temperature change is a gradual transition process in a narrow transition range [*T*_c_ – Δ*T*, *T*_c_ + Δ*T*]. To ensure precise temperature control in subsequent calculations, the dielectric function of VO_2_ is defined as follows [39]:

(1) $$\begin{array}{ll} \varepsilon_{\text{transition}} & =\frac{\varepsilon_{m}+\varepsilon_{i}}{2}+\arctan\left(\frac{T-T_{C}}{\Delta T}\times 10\right)\times \left(\frac{\varepsilon_{m}-\varepsilon_{i}}{2\arctan10}\right), \end{array}$$

where *ɛ*_*m*_ and *ɛ*_*i*_ are denoted as metallic and insulating permittivities, respectively. And the phase-change temperature of VO_2_ is adjusted to *T*_c_ = 20 °C and Δ*T* = 0.1 °C.

**Figure 3:**
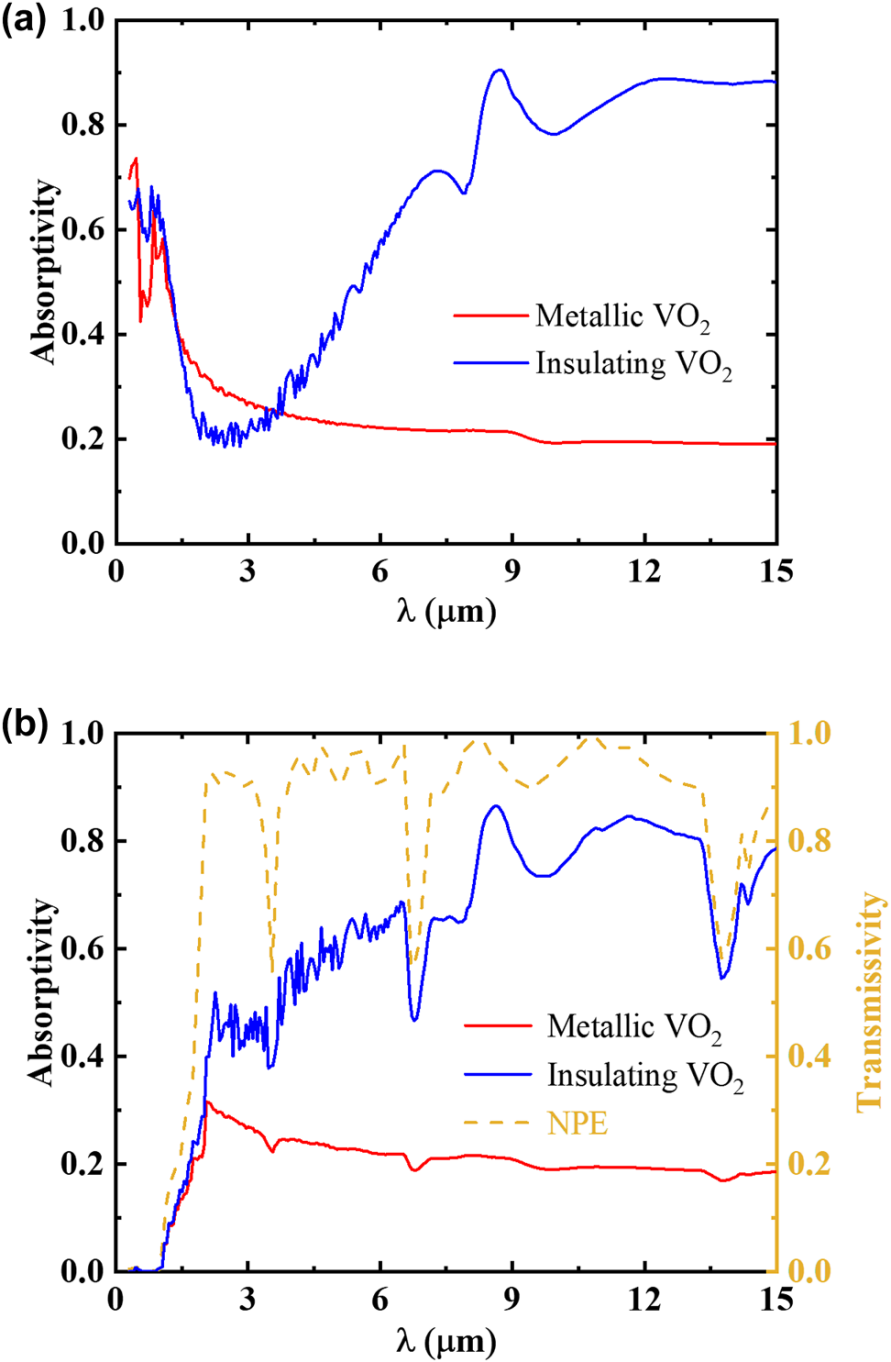
Emissivity of the VO_2_-based radiative cooling system when VO_2_ in metallic and insulating phases (a) without and (b) with the top NPE, respectively, as well as (b) spectral transmissivity of NPE.

The thermal emissivity of the bottom VO_2_-based radiative cooler in the spectral region (*λ* ∼ 0.3–15 μm) is shown in Figure 3(a), where the red curve corresponds to the spectral emissivity of metallic-phase VO_2_, and the blue line represents the spectral emissivity of the insulating-phase VO_2_. It is clearly seen that, near the atmospheric transparent window (*λ* ∼ 8–13 μm), the spectral emissivity of VO_2_-based radiative cooler at different states changes significantly. When the temperature of VO_2_ is below *T*_c_ (insulating-state VO_2_), the infrared emissivity of radiative cooler during the atmospheric window is 0.80. However, as the temperature of VO_2_ rises above *T*_c_ (metallic-state VO_2_), the infrared emissivity decreases substantially to 0.20. The increased temperature of VO_2_-based radiative cooler can significantly suppress infrared emissivity, mainly due to the transition of VO_2_ from insulator to metal around the critical temperature, as well as the associated infrared switching effect. Specifically, when the temperature of VO_2_ is below *T*_c_, the insulating-state VO_2_ thin film exhibits high infrared transmittance. In this state, the emissivity of the VO_2_-based radiative cooler is primarily determined by the broadband-emissive SiO_2_ layer for radiative cooling. However, the VO_2_-based radiative cooler demonstrates high solar absorptivity in both metallic and insulating states, mainly due to the intrinsic lossy characteristics of VO_2_, which is not conducive to achieving significant switchable cooling regulation [38].

To eliminate this drawback, a layer of NPE with high solar reflectance and selective infrared transmission is placed atop the bottom VO_2_ cooler with a certain distance, thereby blocking most solar irradiation from reaching the bottom structure while permitting the passage of most of the infrared radiation from the bottom cooler. Here, we employ incoherent calculations to determine the emissivity for the bottom cooler as follows [38], [56]:

(2) $$\begin{array}{ll} \varepsilon \left(\lambda ,\Omega \right) & =\left(1-r_{C}\right)\left(t_{N}+t_{N}r_{C}r_{N}+t_{N}{\left(r_{C}r_{N}\right)}^{2}\right.\\ & \;\left.+t_{N}{\left(r_{C}r_{N}\right)}^{3}+\cdot \cdot \cdot \right)=t_{N}\left(1-r_{C}\right)/\left(1-r_{C}r_{N}\right) \end{array}$$

where *t*_*N*_ and *r*_*N*_ are the transmissivity and reflectivity of NPE, respectively. And *r*_*C*_ is the reflectivity of the bottom VO_2_-based radiative cooler [56]. After incorporating the top NPE, the emissivity of the composite VO_2_-based cooling system within the same spectral region is depicted in Figure 3(b). Remarkably, the NPE effectively reduces solar absorption when VO_2_ is in both metallic and insulating phases, while maintaining a significant difference in the infrared emissivity between the two phases of VO_2_. This property contributes to create an ideal VO_2_-based cooling system for its auxiliary functions in precise temperature control.

## 3 Results and discussion

When a radiative cooling system operates under a clear sky, the net cooling power *Q*_net_ is determined by solving the thermal balance equation, expressed as follows [6], [38], [57]:

(3) $$\begin{array}{ll} Q_{\text{net}} & =Q_{\text{cooler}}\left(T_{\text{cooler}}\right)-Q_{\text{nr}}\left(T_{\text{amb}},T_{\text{cooler}}\right)-Q_{\text{amb}}\left(T_{\text{amb}}\right)-Q_{\text{sun}}\left(T_{\text{cooler}}\right) \end{array}$$

where *Q*_cooler_ is the radiative power emitted from the radiative cooler, *Q*_nr_ is the nonradiative power, including the heat conduction and convection from the environment, and *Q*_amb_ and *Q*_sun_ represent the atmospheric radiation and sunlight absorbed by the cooler, respectively. Here, *T*_amb_ and *T*_cooler_ are the temperatures of ambient and radiative cooler, respectively. *Q*_cooler_, *Q*_nr_, *Q*_amb_, and *Q*_sun_ can be described as below:

(4) $$Q_{\text{cooler}}\left(T_{\text{cooler}}\right)=A\overset{\infty }{\underset{0}{\int }}d\lambda I_{\text{BB}}\left(T_{\text{cooler}},\lambda \right)\varepsilon \left(\lambda ,\theta ,\phi ,T_{\text{cooler}}\right)$$

(5) $$Q_{\text{nr}}\left(T_{\text{amb}},T_{\text{cooler}}\right)=Ah_{\text{nr}}\left(T_{\text{amb}}-T_{\text{cooler}}\right)$$

(6) $$\begin{array}{ll} & \;Q_{\text{amb}}\left(T_{\text{amb}}\right)=A\overset{\infty }{\underset{0}{\int }}d\lambda I_{\text{BB}}\left(T_{\text{amb}},\lambda \right)\varepsilon \left(\lambda ,\theta ,\phi ,T_{\text{cooler}}\right)\varepsilon \left(\lambda ,\theta ,\phi \right) \end{array}$$

(7) $$Q_{\text{sun}}\left(T_{\text{cooler}}\right)=A\overset{\infty }{\underset{0}{\int }}d\lambda I_{\text{AM}1\text{.}5}\left(\lambda \right)\varepsilon \left(\lambda ,\theta_{\text{sun}},T_{\text{cooler}}\right)$$

where *A* is the surface area of the radiative cooler, $I_{\text{BB}}\left(T_{\text{cooler}},\lambda \right)=2hc^{2}\lambda^{-5}\exp{\left(hc/\lambda k_{\text{B}}T-1\right)}^{-1}$ is the spectral blackbody radiance. In Eq. (7), *I*_AM1.5_(*λ*) represents the spectral irradiance intensity of solar irradiation at AM 1.5, and *ɛ*_atm_(*λ*, *θ*) = 1 − *t*(*λ*)^1/cos*θ*^ is the atmospheric emissivity, in which *t*(*λ*) stands for the atmospheric transmission coefficient in the zenith direction [6]. The nonradiative heat transfer coefficient is donated as *h*_nr_ (0–12 W/m^2^/K), as seen in Eq. (5). Here, *h*_nr_ = 8 W/m^2^/K is used throughout the calculations. A detailed explanation of the formula above can be referred to the literature [38], [58]. Finally, considering the heat transfer from indoor air conditioner to the radiative cooler, the time-dependent temperature of the radiative cooler can be obtained as follows:

(8) $$C_{\text{cooler}}\frac{dT}{dt}=Q_{\text{net}}\left(T_{\text{cooler}},T_{\text{amb}}\right)+Q_{\text{AC}}.$$

where *Q*_AC_ represents the heat transfer from indoor air conditioner to the radiative cooler through heat conduction, and *C*_cooler_ is the heat capacitance of the radiative cooler.

**Figure 4:**
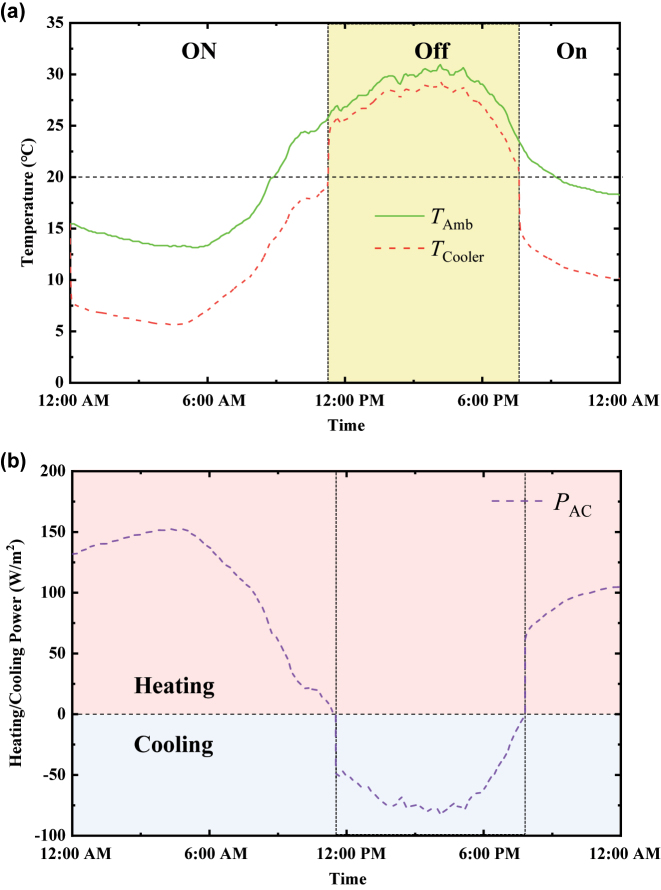
Thermal performance of the radiative cooling system and real-time heating and cooling power of the indoor air conditioner. (a) Temperature response of the VO_2_-based radiative cooling system (red curve) over a 24-h cycle with varying ambient temperature (red curve). (b) Real-time heating and cooling power of the indoor air conditioner required to maintain the wall temperature at a constant 20 °C over the same 24-h period.

Before coupling indoor air conditioning technology, we first analyze the dynamic cooling performance of the VO_2_-based radiative cooling system by calculating its temperature under the typical 24-h outdoor weather condition with large temperature difference between day and night (July 20, 2018, in Stanford, California) [38]. Figure 4(a) displays the temperature variation of the VO_2_-based radiative cooler throughout a 24-h period without using air conditioning. Before 11:00 AM, the VO_2_-based radiative cooler temperature is below the critical temperature 20 °C (*T* < *T*_C_), the insulating-phase VO_2_ enhances the infrared emissivity of VO_2_-based radiative cooler, thereby turning on radiative cooling and creating a large temperature difference below the ambient temperature. Between 11:00 AM and 7:00 PM, when the temperature of the radiative cooler exceeds 20 °C (*T* > *T*_C_), VO_2_ turns to its metallic phase and then closes radiative cooling, which keeps its temperature near the variable ambient temperature. As a result, the single VO_2_-based radiative cooler enhances radiative cooling under low ambient temperatures and suppresses cooling when the ambient temperature is high, thereby functioning as a reverse radiative “thermostat” [41]. This behavior can pose challenges in maintaining stable temperature control for objects. Without accounting for changes in the radiative characteristics of the outdoor VO_2_-based radiative cooler caused by the heat transfer of the indoor air conditioner, Figure 4(b) illustrates the real-time heating and cooling power per area of the air conditioner required to maintain the wall temperature constant at 20 °C over the same 24-h period. Here, for simplification in our calculations, we assume that the wall material is made of high thermal conductivity materials, with no heat loss, and the wall temperature equals the temperature of the VO_2_-based radiative cooler. Figure 4(b) clearly shows that, when the wall temperature falls below the ambient temperature, the indoor air conditioner activates the heating mode, and when the wall temperature rises above the ambient temperature, the indoor air conditioner switches to cooling. After integrating over a 24-h period, the heating and cooling power per unit area of the wall to maintain a stable wall temperature at 20 °C, driven by the indoor air conditioner, amounts to 7,713,629 J/m^2^.

**Figure 5:**
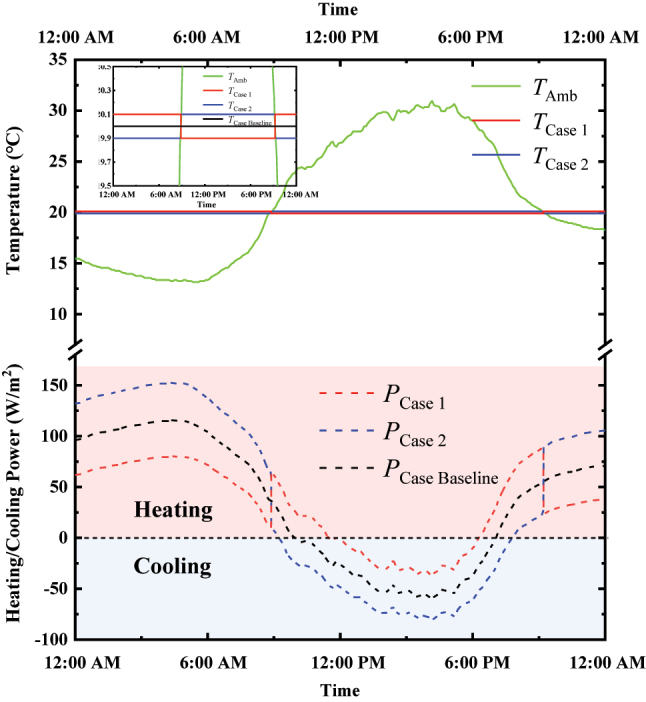
Wall temperature distributions in Case 1 and Case 2, as well as Case Baseline (without VO_2_-based radiative cooling system), along with the corresponding real-time heating and cooling power of the indoor air conditioner to maintain the wall temperature around 20 °C over the same 24-h period.

In fact, different from the calculations mentioned above, in the precise control of wall temperature, the key factor affecting the spectral switching of the VO_2_-based radiative cooler is the indoor air conditioner, rather than the ambient temperature. This is because the atmospheric radiation and the convective heat transfer from the ambient environment are far less significant than the direct cooling and heating provided by the indoor air conditioner. Under these conditions, indoor air conditioner directly affects the temperature of the VO_2_-based radiative cooler through its heating and cooling actions, thereby regulating the infrared emissivity radiative cooler. As a result, both the indoor air conditioner and the outdoor VO_2_-based radiative cooler operate in tandem to achieve temperature stabilization of the wall. As an example in this work, the focus is on precisely controlling the wall temperature around 20 °C. During the process of precise temperature control, the indoor air conditioner will first turn on the heating when the ambient temperature is below 20 °C, then raising the temperature of VO_2_-based radiative cooler to around 20 °C. Conversely, the air conditioner turns on the cooling at high ambient temperatures, thereby reducing the temperature of the VO_2_-based radiative cooler to around 20 °C. In order to play the role of a switchable VO_2_-based radiative cooler in precise temperature control, two different heating and cooling cases for indoor air conditioning will be considered in the following calculations, as shown in Figure 5. Case 1 is that the air conditioner heats the temperature of the VO_2_-based radiative cooler to 20.1 °C when the ambient temperature is below 20 °C and then cools it to 19.9 °C when the ambient temperature is above 20 °C. Case 2 is that the air conditioner heats the radiative cooler temperature to 19.9 °C and cools it to 20.1 °C under the same ambient temperature conditions, respectively. Based on the temperature distribution of VO_2_-based radiative cooler assumed above, the real-time heating and cooling power of the air conditioner over the same 24-h period are calculated based on Eqs. (3)–(8), as seen in Figure 5. It is evident that, regardless of the heating or cooling stage for the indoor air conditioning, Case 2 consumes more energy than Case 1. In Case 1, the cooling operation of the VO_2_-based radiative cooler is synchronized with the switching between heating and cooling modes of the air conditioner. When the air conditioner is in heating mode, the VO_2_-based radiative cooler automatically turns off radiative cooling and then turns on radiative cooling when the air conditioner is in cooling mode. Conversely, in Case 2, the VO_2_-based radiative cooler turns on radiative cooling when the air conditioner is in heating mode and turns off radiative cooling when the air conditioner is in cooling mode, thereby resulting in increased energy consumption by indoor air conditioners. Table 1 gives the detailed parameters of Case 1 and Case 2. Meanwhile, we also calculated the power consumption of the indoor air conditioner without any VO_2_-based radiative cooling system for temperature regulation at 20 °C, referred to as Case Baseline, where the spectral emissivity of exterior wall is calculated by averaging the spectral emissivities of metallic VO_2_ and insulating VO_2_, as shown in Figure 1S. From Figure 5, under the condition of maintaining the wall temperature at 20 °C, the heating and cooling power consumed by indoor air conditioner within 24 h is 5,345,760 J/m^2^ in Case Baseline and 7,335,583 J/m^2^ in Case 2, which is nearly twice the value of Case 1, at approximately 3,782,783 J/m^2^. Another outdoor weather condition on October 3, 2023, in Boston, MA, was also taken into consideration, as shown in Figure 2S. Compared with Case Baseline without switchable radiative cooling, the reverse VO_2_-based radiative cooling system in Case 1 can substantially reduce the energy consumption by nearly 30 % for heating and cooling of indoor air conditioning, as calculated by (5,345,760–3,782,783)/5,345,760 = 30 %. In addition, comparing Case 1 and Case 2, the combination of passive switchable radiative cooling with active indoor air conditioning, coordinating their heating and cooling modes at the same frequency, results in significant energy savings during precise temperature control.

**Table 1:** Wall temperature distributions in Case 1 and Case 2 and the corresponding radiative cooling states of the reverse VO_2_-based radiative cooler over the same 24-h period.

Case	*T*_amb_	*T*_cooler_	Air conditioner	Radiative cooling
Case 1	*T*_amb_ < 20 °C	*T*_cooler_ = 20.1 °C	Heating	Turn off
	*T*_amb_ > 20 °C	*T*_cooler_ = 19.9 °C	Cooling	Turn on
Case 2	*T*_amb_ < 20 °C	*T*_cooler_ = 19.9 °C	Heating	Turn on
	*T*_amb_ > 20 °C	*T*_cooler_ = 20.1 °C	Cooling	Turn off

**Figure 6:**
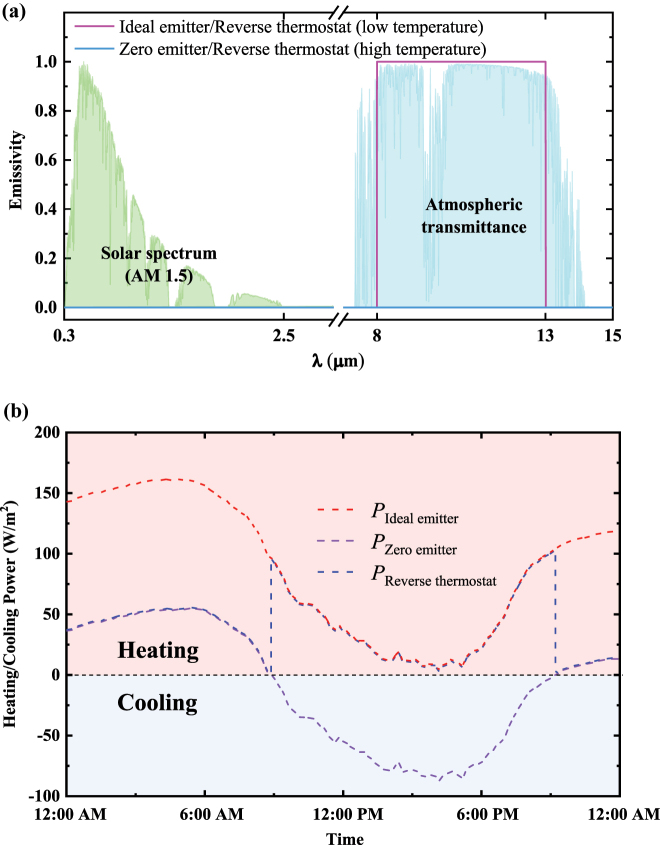
Comparison of three ideal radiators. (a) Emissivity comparison of the ideal emitter with one-value infrared emissivity, the zero emitter with zero-value infrared emissivity, and the ideal reverse thermostat with switchable one-zero-value infrared emissivity. (b) The corresponding real-time heating and cooling power required by the indoor air conditioner to maintain the wall temperature at 20 °C using these three radiators over the same 24-h period.

To further illustrate the energy-saving advantages of passive switchable radiative cooling technology during the precise temperature control dominated by indoor air conditioning, we compare the heating and cooling powers of the indoor air conditioner under three types of ideal radiators installed outside. Three ideal radiators include one-value infrared emissivity (ideal emitter), zero-value infrared emissivity (zero emitter), and switchable one-zero-value infrared emissivity (reverse thermostat). The spectral emissivity distributions for these radiators are shown in Figure 6(a). In this case, the ideal reverse thermostat exhibits an infrared thermal emissivity of 0 within the atmospheric transparent window (8–13 μm) at high temperature, while having an emissivity of 1 at low temperature. Similarly, the real-time heating and cooling powers of the air conditioner, maintaining the wall temperature around 20 °C, over the same 24-h period are calculated based on these three ideal radiators, as seen in Figures 6(b) and 3S. The heating and cooling powers used by the indoor air conditioner within 24 h are as follows: 7,705,658 J/m^2^ (ideal emitter), 3,863,389 J/m^2^ (zero emitter), and 3,300,983 J/m^2^ (reverse thermostat), respectively. While the ideal emitter can maximize the radiative cooling power at higher ambient temperatures and the zero emitter can minimize the radiative cooling power at lower ambient temperatures, the energy expended by the air conditioning system with the ideal reverse thermostat remains the lowest among the daily accumulated energy consumption. This is due to the reverse thermostat synchronizes its cooling modes with the heating and cooling cycles of the indoor air conditioning, leading to minimal energy consumption by the indoor air conditioning system. Therefore, the above comparison further demonstrates that reverse radiative cooling system can effectively assist indoor air conditioners in reducing energy consumption during precise temperature control.

## 4 Conclusions

In conclusion, we propose and analyze a reverse-switching VO_2_-based radiative cooling system, assisting indoor air conditioning to achieve precise indoor temperature control. Different from previous VO_2_-based radiative cooling systems, our proposed VO_2_-based radiative cooler automatically turns on radiative cooling at low ambient temperatures and turns off radiative cooling at high ambient temperatures. Rather than independently adjusting its infrared emissivity to stabilize indoor temperatures, the passive VO_2_-based radiative cooling system synchronizes its cooling modes with the heating and cooling cycles of the indoor air conditioning during the real-life process of precise temperature control. The calculation results demonstrate that compared with the baseline system without switchable radiative cooling, the reverse VO_2_-based radiative cooling system can substantially reduce the energy consumption by nearly 30 % for heating and cooling of indoor air conditioning while maintaining a constant indoor temperature, outperforming even the ideal radiative cooler. The precise temperature control is vital for achieving specific outcomes, maintaining product quality, ensuring equipment safety, and enhancing the human comfort. Therefore, this work will provide a potential application approach for radiative cooling technology and advance its intelligent thermal regulation alongside traditional air conditioning technology.

## Supplementary Material

Supplementary Material Details
