# Supplementary material for: Reverse-switching radiative cooling for synchronizing indoor air conditioning
Source: Nanophotonics. 2024 Feb 1;13(5):701–10. doi: 10.1515/nanoph-2023-0699 (PMC11501576; doi:10.1515/nanoph-2023-0699)
Supplement: Supplementary file 1 — Supplementary Material Details [file j_nanoph-2023-0699_suppl_001.docx]

Supplementary material

**Reverse-switching radiative cooling for synchronizing indoor air conditioning**

Yang Liu ^a^ and Yi Zheng ^a,b∗^

*^a^Department of Mechanical and Industrial Engineering, Northeastern University, Boston, MA 02115, USA*

*^b^Department of Chemical Engineering, Northeastern University, Boston, MA 02115, USA*

*Electronic mail: y.zheng@northeastern.edu

**Figure 1S:** Emissivity of the VO_2_-based radiative cooling system when VO_2_ in metallic and insulating phases with the top NPE, as well as the baseline system calculated by averaging the spectral emissivities of metallic VO_2_ and insulating VO_2_.

**Figure 2S:** Wall temperature distributions in Case 1 and Case 2, as well as Case Baseline (without VO_2_-based radiative cooling system), along with the corresponding real-time heating and cooling power of the indoor air conditioner to maintain the wall temperature around 20 ℃ over another 24-hour outdoor weather condition (October 3, 2023, in Boston, MA).

**Figure 3S:** Comparison of the real-time heating and cooling power required by the indoor air conditioner to maintain the wall temperature at 20 ℃ using the actual VO_2_-based reverse-thermostat and the ideal reverse-thermostat over the same 24-hour period.
